# Supplementary material for: Dupilumab Efficacy on Asthma Functional, Inflammatory, and Patient-Reported Outcomes across Different Disease Phenotypes and Severity: A Real-Life Perspective
Source: Biomedicines. 2024 Feb 8;12(2):390. doi: 10.3390/biomedicines12020390 (PMC10886692; doi:10.3390/biomedicines12020390)

Figure S1: Trend of lung function parameters over dupilumab treatment, including individual data plotting. Time on X-axis is expressed in months. The grey area indicates the 0.95 confidential interval

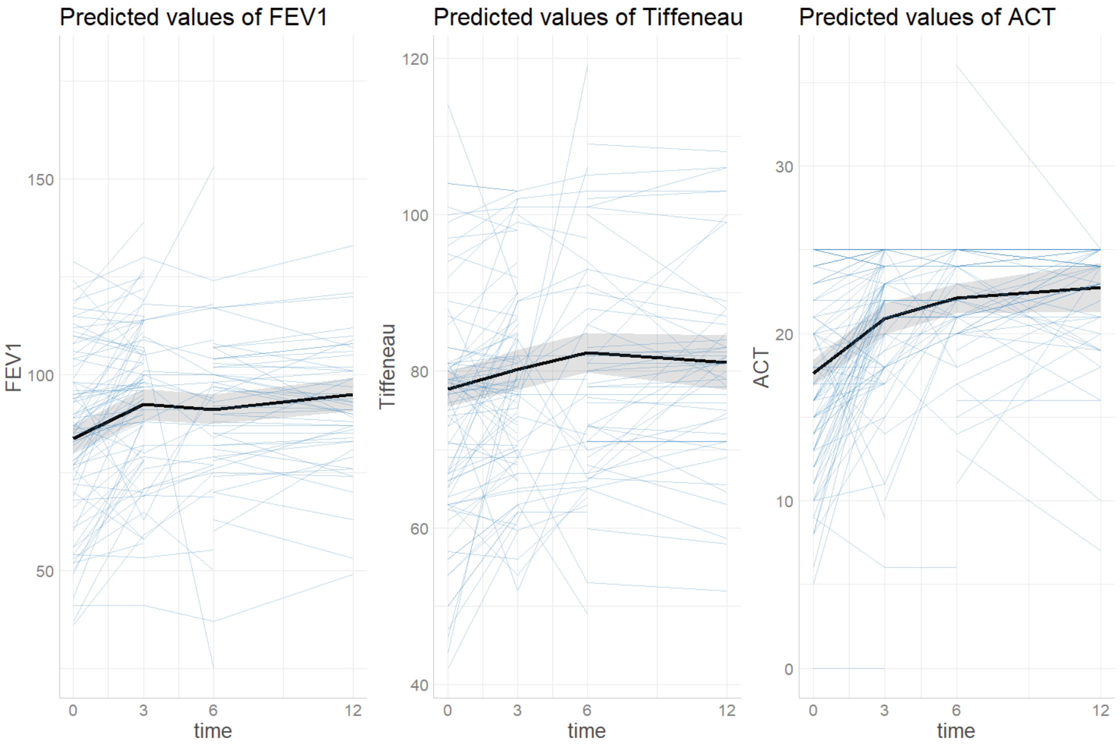

Figure S2: Change of different asthma-related outcomes during dupilumab treatment, including individual data plotting. OCS= oral corticosteroids. Time on X-axis is expressed in months. The grey area indicates the 0.95 confidential interval

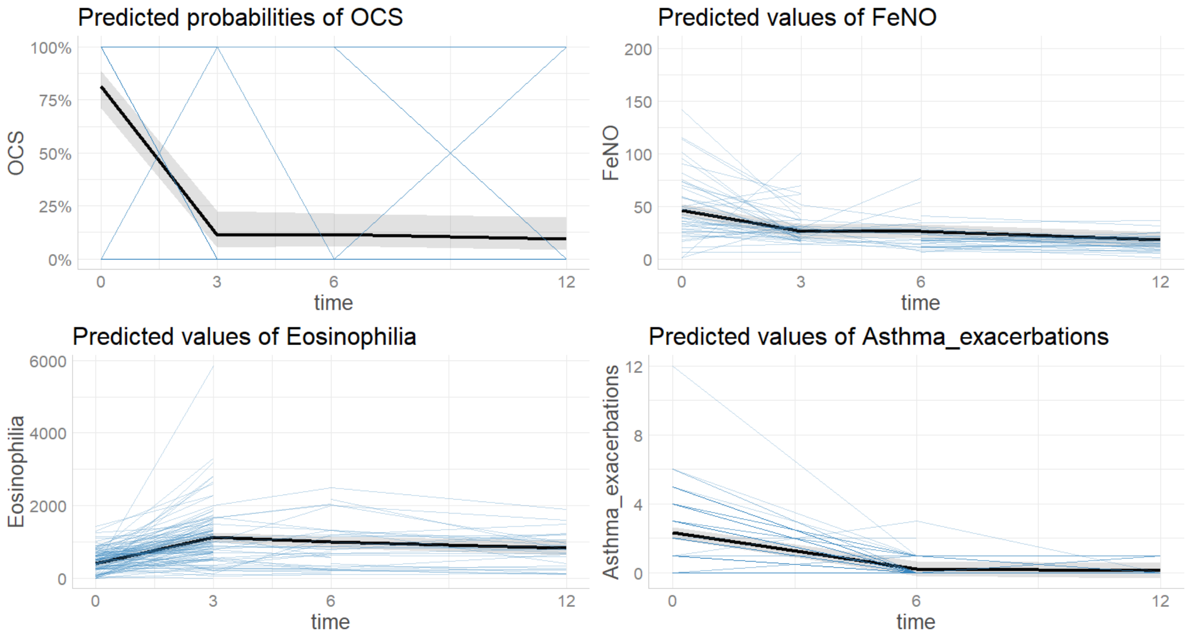

Figure S3: Trend of nasal polyps related outcomes over dupilumab treatment, including individual data plotting. Time on X-axis is expressed in months. The grey area indicates the 0.95 confidential interval. SNOT22= sinonasal outcome test; VAS= visual analogic scale; NPS= nasal polyp score.

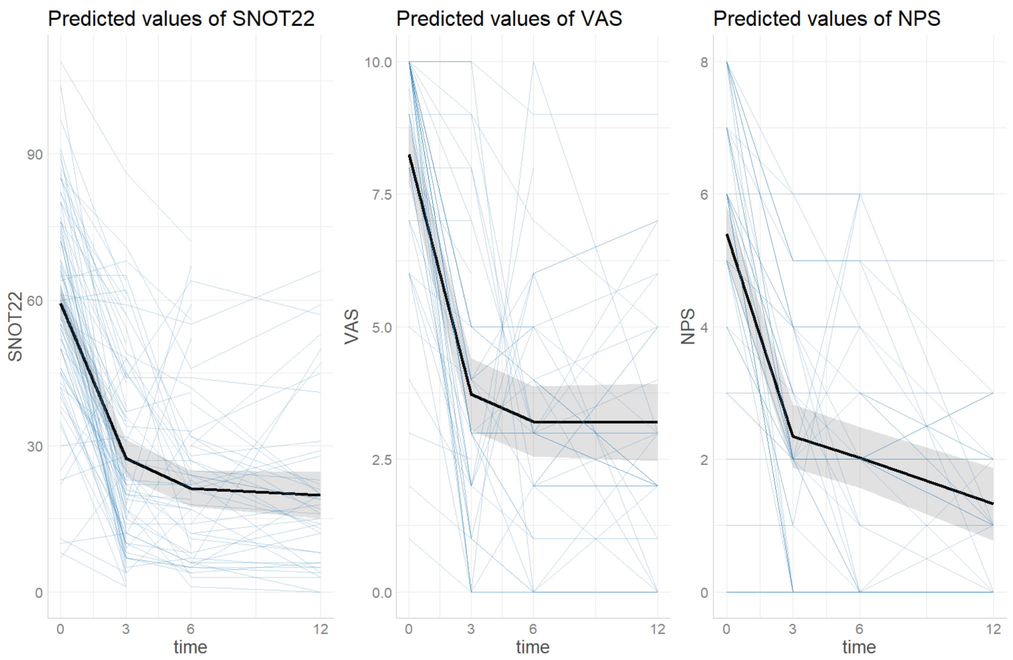

Supplement: Supplementary file 1 [file biomedicines-12-00390-s001.zip › biomedicines-2827126-supplementary -revised.pdf]
